# The Importance of Surface Adsorbates in Solution‐Processed Thermoelectric Materials: The Case of SnSe

**DOI:** 10.1002/adma.202106858

**Published:** 2021-10-22

**Authors:** Yu Liu, Mariano Calcabrini, Yuan Yu, Aziz Genç, Cheng Chang, Tommaso Costanzo, Tobias Kleinhanns, Seungho Lee, Jordi Llorca, Oana Cojocaru‐Mirédin, Maria Ibáñez

**Affiliations:** ^1^ IST Austria Am Campus 1 Klosterneuburg 3400 Austria; ^2^ RWTH Aachen I. Physikalisches Institut (IA) Sommerfeldstraße 14 52074 Aachen Germany; ^3^ Department of Materials Science and Engineering Faculty of Engineering İzmir Institute of Technology İzmir 35430 Turkey; ^4^ Institute of Energy Technologies Department of Chemical Engineering and Barcelona Research Center in Multiscale Science and Engineering Universitat Politècnica de Catalunya Barcelona 08019 Spain

**Keywords:** grain boundaries, liquid sintering, SnSe, solution processing, surface chemistry, thermoelectricity

## Abstract

Solution synthesis of particles emerges as an alternative to prepare thermoelectric materials with less demanding processing conditions than conventional solid‐state synthetic methods. However, solution synthesis generally involves the presence of additional molecules or ions belonging to the precursors or added to enable solubility and/or regulate nucleation and growth. These molecules or ions can end up in the particles as surface adsorbates and interfere in the material properties. This work demonstrates that ionic adsorbates, in particular Na^+^ ions, are electrostatically adsorbed in SnSe particles synthesized in water and play a crucial role not only in directing the material nano/microstructure but also in determining the transport properties of the consolidated material. In dense pellets prepared by sintering SnSe particles, Na remains within the crystal lattice as dopant, in dislocations, precipitates, and forming grain boundary complexions. These results highlight the importance of considering all the possible unintentional impurities to establish proper structure–property relationships and control material properties in solution‐processed thermoelectric materials.

## Graphical abstract

Polycrystalline SnSe is prepared from surfactant‐free particles prepared in solution. Ionic species adsorbed on the surface compensate for the particles′ charge and remain during processing. The adsorbates affect the nano/microstructure of the final material and its transport properties. In the case of sodium, it remains as a dopant and forms secondary phases that induce energy‐filtering effects.

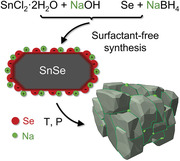

## 1 Introduction

Thermoelectric devices reversibly convert heat into electricity both for power harvesting and for active cooling and heating. The efficiency of a thermoelectric device is determined by the temperature‐dependent properties of the materials that constitute it. These are summarized in the thermoelectric figure of merit of the material, *zT* = *S* ^2^ *σκ* ^–1^ *T*, that combines Seebeck coefficient (*S*), electrical conductivity (σ), thermal conductivity (κ), and absolute temperature (*T*).^[^ ^1^, ^2^ ^]^

Thermoelectric materials are often dense, polycrystalline inorganic semiconductors. Usually, the processing of such materials has two steps: preparation of the semiconductor in powder form and the consolidation of the powder into a dense sample. The most common route to prepare powders among the thermoelectric community is through high‐temperature reactions and ball milling.^[^ ^3^ ^]^ Alternatively, solution methods to produce powders with much less demanding conditions (e.g., lower reagent purity, lower temperatures, and shorter reaction times) have been explored to reduce the production costs.^[^ ^1^, ^4^ ^]^ These methods also provide opportunities to produce particles with better‐controlled features, such as crystallite size, shape, composition, and crystal phase, which allow modifying the properties of the consolidated material.^[^ ^5^, ^6^, ^7^ ^]^ Lastly, solution processing facilitates device fabrication versatility, including flexible, conformable, and rigid modules with customized geometries.^[^ ^8^, ^9^ ^]^ To date, solution synthesis has enabled the production of several materials with state‐of‐the‐art performances, as is the case of PbS,^[^ ^6^ ^]^ Bi_0.5_Sb_1.5_Te_3_,^[^ ^10^ ^]^ Bi_2_Te_3−_ *_x_* Se*_x_*,^[^ ^11^ ^]^ and SnTe,^[^ ^12^ ^]^ demonstrating the potential of this strategy.

However, when dealing with powders produced in solution, one should pay special attention to potential undesired elements coming from the reactants. Those elements may not affect the crystal structure and bulk composition of the powder but can be present as surface adsorbates.^[^ ^13^ ^]^ The composition, chemical stability, and bonding nature of surface species can influence the sintering process, and reaction byproducts can determine the final properties of the consolidated material. Surface species have been carefully considered in surfactant‐assisted colloidal synthesis because of the insulating nature of the long‐chain aliphatic surfactants generally used^[^ ^14^ ^]^ and the consequent detrimental effects on thermoelectric performance.^[^ ^15^ ^]^ In such cases, the surfactants have to be removed to enhance the electrical conductivity. The most common strategy to do so is thermal decomposition.^[^ ^16^, ^17^, ^18^ ^]^ Alternatively, surfactants can be exchanged with volatile compounds^[^ ^19^ ^]^ or even inorganic species^[^ ^20^ ^]^ that can further tune material properties.^[^ ^12^, ^18^, ^21^ ^]^ Conversely, the presence of surface adsorbates is usually neglected in the case of the so‐called surfactant‐free methods,^[^ ^4^, ^22^, ^23^, ^24^, ^25^, ^26^, ^27^, ^28^, ^29^, ^30^, ^31^, ^32^, ^33^, ^34^ ^]^ the most widely used to produce thermoelectric powders in solution. The vast majority of reports dealing with surfactant‐free synthesis do not perform any surface treatments since the particles are considered “naked”.^[^ ^32^, ^35^, ^36^ ^]^ This is a misconception since different species might be adsorbed on the particle surface, depending on the particle composition and surface termination.^[^ ^37^, ^38^, ^39^, ^40^ ^]^ Such adsorbates need to be identified to unveil if they have a role in the thermoelectric properties.

In this work, we identify the surface species resulting from the aqueous synthesis of SnSe particles and demonstrate their effects on the microstructure and thermoelectric properties of the final material. While this work focuses on SnSe, one of the most studied thermoelectric materials due to its high performance, the presence of surface species in solution‐processed materials goes beyond the specific material system and needs to be carefully evaluated to understand the material properties correctly.

## 2 Results and Discussion

### 2.1 Material Processing: From Particle Synthesis to Pellet Formation

The table below shows the most common reactants to synthesize SnSe powders in solution that lead to state‐of‐the‐art performances (**Table** **1**). SnSe particles are usually prepared in polar media (water or ethylene glycol, EG) using SnCl_2_ and Se, SeO_2_, or Na_2_SeO_3_ as precursors. Additionally, redox agents and acids or bases are used. Generally, these chemicals are Na salts, as highlighted in bold in Table 1.

**Table 1:** Summary of the state‐of‐art thermoelectric performance of p‐type doped polycrystalline SnSe prepared by consolidating solution‐processed particles. The table includes the reported composition of the consolidated material, the reactants used for the synthesis, and peak *zT* values (*zT*_max_) at the corresponding temperature. “||” and “⊥” denote the direction in which those results were obtained; “||” refers to the direction parallel to the pressing axis and “⊥” refers to the perpendicular one

Material	Precursors, solvent	*zT*_max_
SnSe‐3%CdSe^[^ ^41^ ^]^	SnCl_2_·2H_2_O, **NaBH_4_**, **NaOH**, Se, CdO, H_2_O	2.2 (786 K, ||)
Sn_0.96_Ga_0.04_Se^[^ ^22^ ^]^	SnCl_2_·2H_2_O, GaCl_3_, **NaOH**, Se, H_2_O	2.2 (873 K, ||)
Sn_0.98_Pb_0.01_Zn_0.01_Se^[^ ^29^ ^]^	SnCl_2_·2H_2_O, PbCl_2_, ZnCl_2_, **NaOH,** Se, H_2_O	2.2 (873 K, ||)
Sn_0.95_Se^[^ ^27^ ^]^	SnCl_2_·2H_2_O, **NaOH**, Se, H_2_O	2.1 (873 K, ||)
Sn_0.97_Ge_0.03_Se^[^ ^4^ ^]^	SnCl_2_·2H_2_O, GeI_4_, **NaOH**, Se, H_2_O	2.1 (873 K, ⊥)
Sn_0.99_Pb_0.01_Se–Se QDs^[^ ^42^ ^]^	SnCl_2_·2H_2_O, PbCl_2_, **NaOH**, Se, H_2_O	2.0 (873 K, ||)
Sn_0.96_Pb_0.01_Cd_0.03_Se^[^ ^24^ ^]^	SnCl_2_·2H_2_O, PbCl_2_, CdCl_2_, **NaOH**, Se, H_2_O	1.9 (873 K, ||)
Sn_0.948_Cd_0.023_Se^[^ ^43^ ^]^	SnCl_2_·2H_2_O, CdCl_2_, **Na_2_SeO_3_**, **NaOH**, EG	1.7 (823 K, ⊥)
SnSe‐4%InSe_y_^[^ ^44^ ^]^	SnCl_2_·2H_2_O, InCl_3_·4H_2_O, **Na_2_SeO_3_**, **NaOH**, EG	1.7 (823 K, ⊥)
SnSe‐1%PbSe^[^ ^25^ ^]^	SnCl_2_·2H_2_O, PbCl_2_, **NaOH**, Se, H_2_O	1.7 (873 K, ||)
NaOH‐Sn_1−_ *_x_* Se^[^ ^30^ ^]^	SnCl_2_, **Na_2_SeO_3_**, **NaOH**, EG	1.5 (823 K, ⊥)
Sn_0.882_Cu_0.118_Se^[^ ^34^ ^]^	SnCl_2_·2H_2_O, CuCl_2_, **Na_2_SeO_3_**, **NaOH**, EG	1.41 (823 K, ⊥)
SnSe‐15%Te NWs^[^ ^45^ ^]^	SnCl_2_·2H_2_O, **NaBH_4_**, **NaOH**, Se, H_2_O	1.4 (790 K, ||)
Sn_0.98_Se^[^ ^31^ ^]^	SnCl_2_·2H_2_O, **Na_2_SeO_3_**, **NaOH**, EG	1.36 (823 K, ⊥)
SnSe_0.90_Te_0.1_^[^ ^46^ ^]^	SnCl_2_·2H_2_O, **Na_2_SeO_3_**, **Na_2_TeO_3_**, **NaOH**, EG	1.1 (800 K, ⊥)
Sn_0.99_Cu_0.01_Se^[^ ^28^ ^]^	SnCl_2_·2H_2_O, CuCl, **NaOH**, Se, H_2_O	1.2 (873 K, ||)
SnSe_0.9_S_0.1_^[^ ^23^ ^]^	SnCl_2_·2H_2_O, **Na_2_S**, **NaBH_4_**, **NaOH**, Se, H_2_O	1.16 (923 K, ⊥)

**Figure 1:**
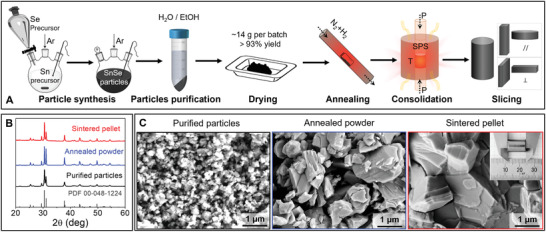
A) Scheme of the processing steps of SnSe pellets. B) XRD patterns and C) the corresponding representative SEM micrographs of the initial SnSe particles, the annealed powder, and the sintered pellet along the out‐of‐plane direction. The inset in figure (C) shows a picture of a dense SnSe cylindrical pellet.

Herein, we selected the simplest and most cost‐effective synthetic method, which uses ambient pressure and water as the solvent,^[^ ^33^ ^]^ as a prototypical reaction. In the chosen procedure, NaBH_4_ is first dissolved in water, and Se powder is slowly added to form HSe^−^. In parallel, NaOH and SnCl_2_·2H_2_O are dissolved in water, and the solution is heated to its boiling point. At this temperature, the freshly prepared Se‐solution is rapidly injected. Upon injection, the reaction mixture turns black, indicating particle formation, and is kept under reflux for an additional 2 h. The as‐synthetized particles are purified by precipitation/redispersion with water and ethanol and then dried under vacuum overnight at room temperature. Afterward, the powder is annealed in forming gas (95% N_2_ + 5% H_2_) to remove oxide species.^[^ ^47^ ^]^ Finally, the annealed powders are consolidated into cylindrical pellets using spark plasma sintering (SPS). Throughout the process, X‐ray diffraction (XRD) is used to verify that the product is pure phase orthorhombic SnSe (PDF 00‐048‐1224) and scanning electron microscopy (SEM) is used to evaluate the material morphology (**Figure** **1**). The experimental details, from particle synthesis to pellet formation, can be found in the Experimental Section.

### 2.2 Particle Properties

**Figure 2:**
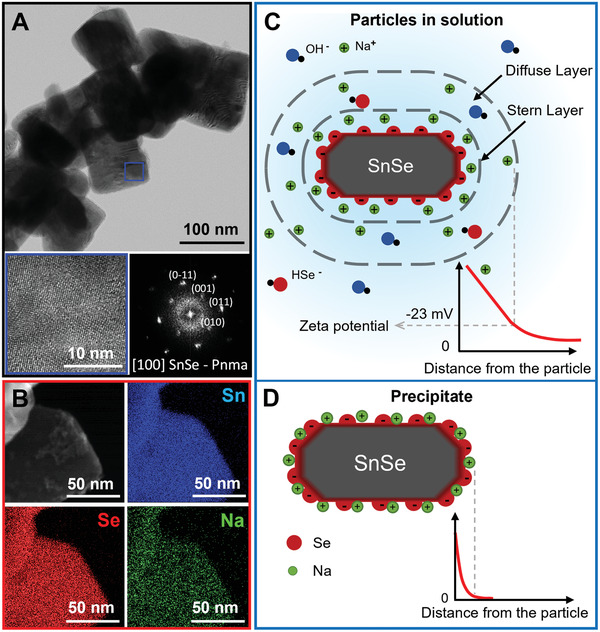
A) Bright‐field TEM micrograph of several SnSe particles, HRTEM micrograph of the region marked with a blue square, and its corresponding power spectrum; B) STEM–EDS elemental mapping of Sn (blue), Se (red), and Na (green) for SnSe particles; C) Schematic representation of the electrical double layer of a SnSe particle in solution based on the Stern model; D) Schematic representation of a precipitated particle with Na^+^ ions adsorbed to ensure charge neutrality.

SEM and transmission electron microscopy (TEM) images show that the particles have a rectangular shape with an average lateral size of ≈150 ± 50 nm (Figures 1C and **2**A). High‐resolution TEM (HRTEM) analysis confirmed the SnSe orthorhombic structure (space group *Pnma*) with lattice parameters *a* = 11.5156 Å, *b* = 4.1571 Å, and *c* = 4.4302 Å. Elemental analysis with energy‐dispersive X‐ray spectroscopy (EDS) in SEM and scanning transmission electron microscopy (STEM) indicated that the particles are slightly Se‐rich (Figure S1, Supporting Information) and revealed the presence of Na (**Figure** **2**B). The Na to Sn ratio determined by X‐ray photoemission spectroscopy (XPS, Figure S2, Supporting Information) is five times larger than that obtained from EDS (Figure S1, Supporting Information), suggesting that Na is mainly at the particle surface, yet we cannot discard its presence within the SnSe particles. In the Se 3d XPS region, four peaks can be deconvoluted. These correspond to the 3d_3/2_ and 3d_5/2_ emission peaks of Se in two different oxidation states. Se^2−^, from SnSe, is observed at low binding energies, while at higher energies, more oxidized Se species are present, with an oxidation state closer to 0.^[^ ^48^ ^]^ We speculate that these species are polyselenides (Se*_x_* ^2−^), formed by partial oxidation of the particle surface during washing, as observed for Cu_2_Se nanoparticles.^[^ ^49^, ^50^, ^51^ ^]^ During the synthesis, Na^+^ ions compensate the charge of the negative ions (OH^−^, BH_4_^−^, HSe^−^, etc.) but should not react based on the chemical equation.^[^ ^33^ ^]^ Therefore, we propose that Na^+^ ions are adsorbed on the surface, explaining their presence in the particles. To verify this hypothesis and disclose the nature of the adsorption, we performed electrophoretic mobility measurements and determined that SnSe particles are negatively charged (zeta potential = −22 ± 5 mV, Figure 2C) consistent with their Se‐rich surface. Based on the electric double layer description, the negatively charged SnSe particles are surrounded by an immobile layer, the Stern layer, of cations (Na^+^) and a second layer of loosely bound ions, the diffuse layer, composed mostly of cations (Na^+^) and some anions (OHˉ, HSeˉ).^[^ ^52^ ^]^ Due to the electrostatic force, cations remain on the particle surface during washing, explaining the presence of Na once the particles are removed from the solution (Figure 2D).

### 2.3 Pellets Microstructure

Compositional analysis with inductively coupled plasma – optical emission spectroscopy (ICP‐OES) and EDS revealed that the pellets have ≈1.6 at% Na content (Figure S1, Supporting Information). Such large Na content, beyond its solubility limit,^[^ ^53^ ^]^ confirms that Na^+^ ions are adsorbed on the particle surface.

**Figure 3:**
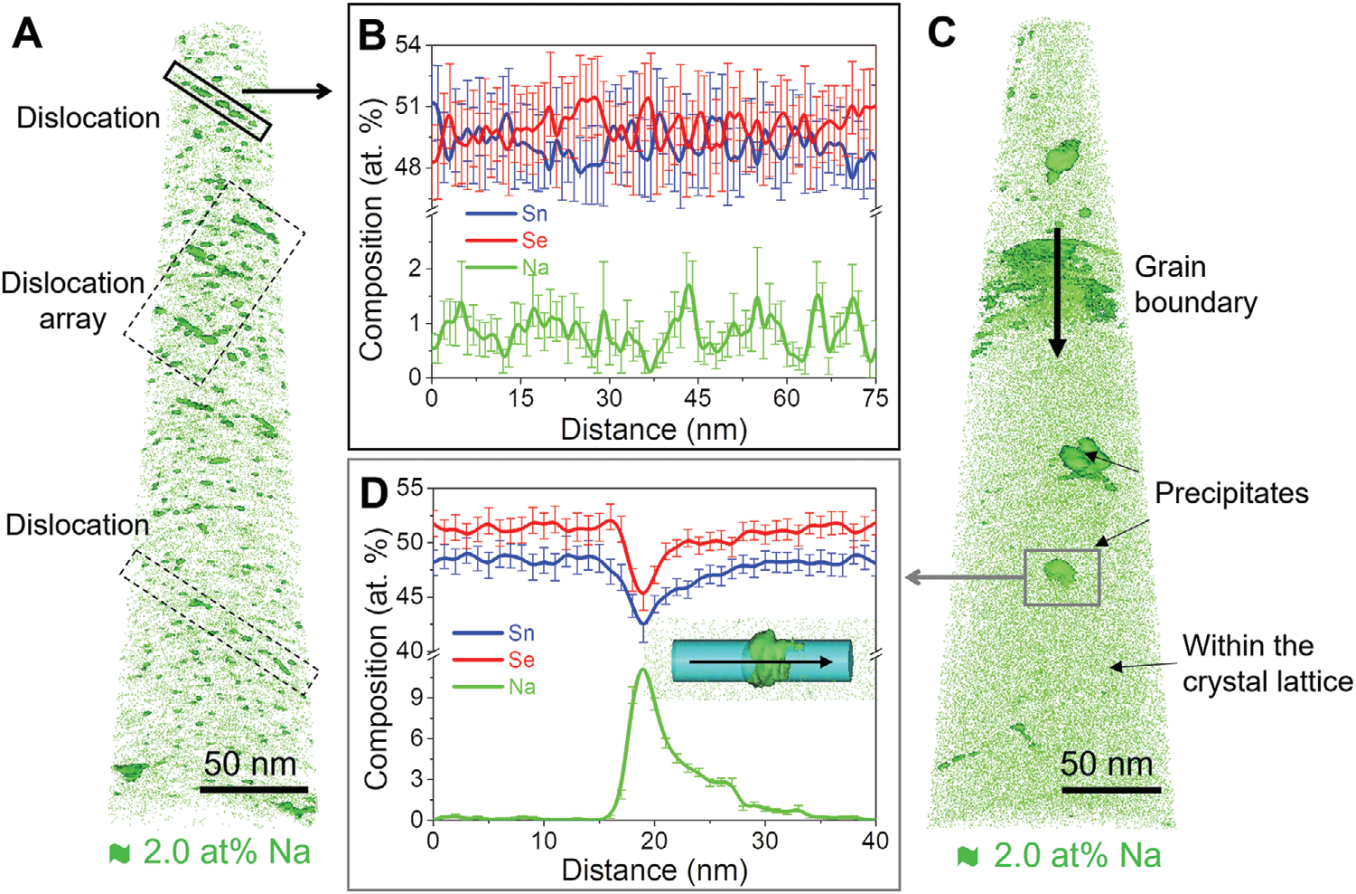
APT of a Na‐containing SnSe pellet. A) 3D distribution of Na showing multiple dislocations highlighted by the Na‐2.0 at% isocomposition surface and indicated by rectangles; B) composition profile along a dislocation showing the presence of Na at dislocation cores; C) 3D distribution of Na in the same pellet showing Na‐rich precipitates, a larger precipitate is observed at the grain boundary;^[^ ^41^ ^]^ D) composition profile across a precipitate.

**Figure 4:**
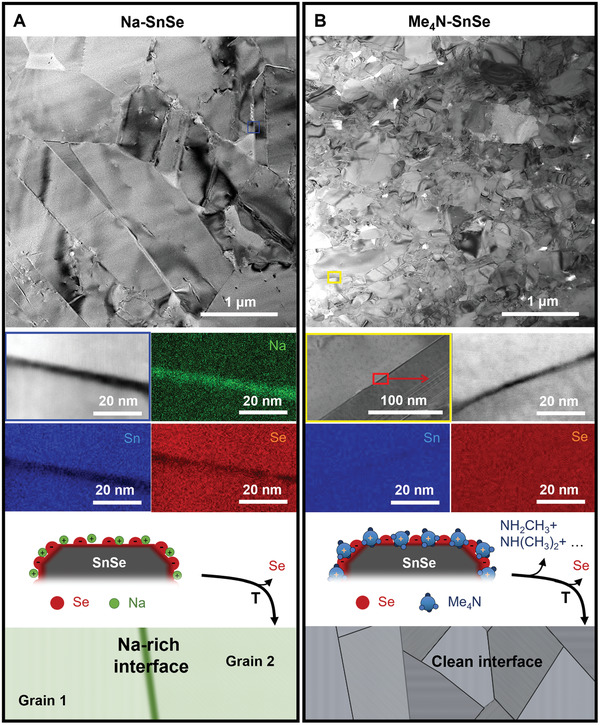
TEM images representing cross‐sections of SnSe pellets. General view (top), together with the corresponding STEM–EDS elemental mapping of a grain boundary (middle), and a scheme of the grain boundary interface (bottom) resulting for the two different cations: A) Na^+^; and B) Me_4_N^+^.

We studied the pellet's microstructure using atom probe tomography^[^ ^54^, ^55^ ^]^ (APT, **Figure** **3**; Figure S3, Supporting Information) and STEM (**Figure** **4**) to determine the distribution of Na in the sintered material. Figure 3 shows the 3D distribution of Na in the pellet illustrated by the 2.0 at% Na isocomposition surfaces. These contour plots delimit the areas where the concentration of Na is ≥2 at%.^[^ ^56^ ^]^ We observe that in the pellet, Na is distributed in four different environments: i) within the crystal lattice; ii) at dislocations; iii) forming grain boundary complexions;^[^ ^57^ ^]^ and iv) in nanoprecipitates^[^ ^41^ ^]^ (Figure 3).

The large number of dislocations (Figure S9, Supporting Information) can be correlated to the large amount of Sn vacancies in the particles and the annealed powder.^[^ ^58^ ^]^ Upon annealing, these vacancies diffuse, creating vacancy aggregates of lower energy that collapse into dislocations.^[^ ^59^, ^60^, ^61^ ^]^ The composition profile along a dislocation line in Figure 3A shows a periodic fluctuation of Na concentration, suggesting that the formation of these dislocations is not only an efficient pathway to relax epitaxial strain but also triggers Na segregation.^[^ ^62^, ^63^ ^]^ Moreover, the large number of defects in polycrystalline SnSe provides heterogeneous nucleation sites for precipitation,^[^ ^64^, ^65^, ^66^ ^]^ explaining the presence of Na‐rich precipitates within the grains, at the dislocations, and in grain boundaries (Figure 3).^[^ ^67^ ^]^ However, we could not identify the exact composition nor crystal structure of the Na‐rich phase due to its high air sensitivity, ease of oxidation, and instability under the electron beam.

When present in the reaction mixture, Na^+^ ions end up on the particle surface and become an involuntary impurity of the consolidated material, referred to as Na–SnSe from now on. To evaluate the effect of Na in the material's microstructure and transport properties, we developed a new synthetic route to obtain Na‐free SnSe particles. In this synthesis, we replaced Na^+^ for a cation that decomposes during the annealing step. In particular, we used tetramethylammonium salts: Me_4_NBH_4_ and Me_4_NOH, instead of NaBH_4_ and NaOH. Following the same synthetic and purification processes, we obtained pure phase SnSe particles (Figure S4, Supporting Information), referred to as Me_4_N–SnSe. XPS analysis of Me_4_N–SnSe particles revealed N 1s emission peaks which can be assigned to tetramethylammonium (Figure S2, Supporting Information).^[^ ^68^ ^]^ None of the elemental analysis techniques used detected Na (Figure S1, Supporting Information). Similar to Na–SnSe, Me_4_N–SnSe particles show a negative surface charge (zeta potential = −23 ± 5 mV). Based on the above, we conclude that the charge balancing ions are Me_4_N^+^ as depicted in the scheme in Figure 4B. We verified the decomposition of adsorbed Me_4_N^+^ by in situ mass spectrometry analysis (Figure S5, Supporting Information), which confirmed the desorption of the organic species at the annealing temperature used, 500 °C, in accordance with previous works.^[^ ^69^ ^]^ However, by APT and Raman spectroscopy, we detected minor traces of N and C in the final pellet (Figures S6 and S7, Supporting Information). XRD patterns of the Me_4_N–SnSe particles, annealed powder, and final pellet show the same diffraction peaks as Na–SnSe (Figure S4, Supporting Information).

Although both synthetic strategies yield SnSe particles with analogous structural properties, the average grain size of the final pellet is much smaller for Me_4_N–SnSe than for Na–SnSe (Figure 4) despite both having the same density. These results show that the adsorbed ions strongly influence the microstructure of the samples. We propose different reasons for the distinct microstructure. First, the aqueous synthesis with Me_4_N^+^ already yields smaller SnSe particles. While Me_4_N^+^ and Na^+^ have the same charge, they differ significantly in size, Me_4_N^+^ having a radius of 322 pm and Na^+^, 98 pm. The larger size of Me_4_N^+^ allows a smaller number of Me_4_N^+^ to fit in the Stern layer, increasing the electrostatic repulsion between the particles and therefore yielding smaller particles.^[^ ^70^ ^]^ Moreover, during the thermal processing, the Se‐rich surface reacts with Na producing sodium polyselenides (Na_2_Se*_x_*), which through melting provide a capillary force that pulls the grains together (Figure S10, Supporting Information).^[^ ^71^, ^72^ ^]^ Finally, the liquid phase facilitates atomic diffusion between the grains and promotes grain growth.^[^ ^72^ ^]^ The presence of low melting point Na_2_Se*_x_* phases as grain growth promoters has been previously reported in chalcogenide solar cell absorbers.^[^ ^73^, ^74^, ^75^ ^]^ In the case of Me_4_N–SnSe, Me_4_N^+^ decomposes into volatile species,^[^ ^76^ ^]^ circumventing the formation of Na_2_Se*_x_* and its effects in grain growth during the thermal processing.

The presence of different cations during the material processing also influences the nature of the grain boundaries. STEM‐EDS analysis across grain boundaries (Figure 4) shows Na‐rich grain boundary complexions in Na–SnSe while Me_4_N–SnSe has clean interfaces.

### 2.4 Transport Properties

**Figure 5:**
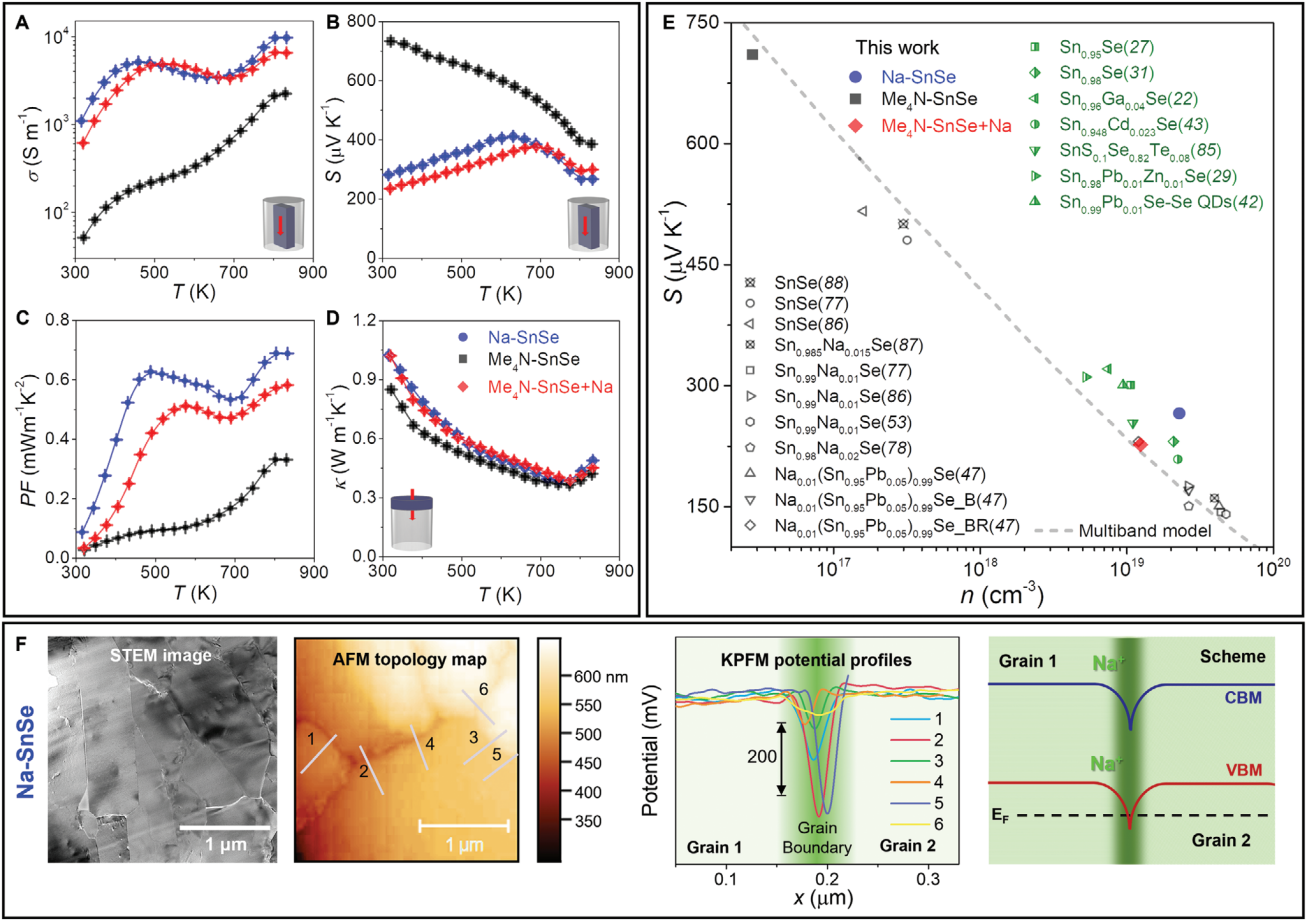
A) Electrical conductivity, σ; B) Seebeck coefficient, *S*; C) power factor, *PF*; D) thermal conductivity, κ; Na–SnSe, Me_4_N–SnSe, and Me_4_N–SnSe + Na samples measured in the direction parallel to the pressing axis. E) Pisarenko plot at 300 K. Green dots are references from solution‐processed materials^[^ ^22^, ^27^, ^29^, ^31^, ^42^, ^43^, ^85^ ^]^ and black dots from solid‐state synthetic methods,^[^ ^47^, ^53^, ^77^, ^78^, ^86^ ^]^ including single crystals.^[^ ^87^, ^88^ ^]^ The dashed line was calculated using a multiple band model.^[^ ^84^ ^]^ F) STEM image, atom probe microscopy (AFM) topology maps, KPFM potential profiles across the lines indicated in the AFM topology map, and scheme showing the band bending at the grain boundary in Na–SnSe. CBM, VBM, and *E*_F_ indicate the conduction band minimum, valence band maximum, and Fermi level, respectively.

To evaluate the effects of the Na–SnSe and Me_4_N–SnSe distinct microstructure and composition in the transport properties, we measured the electrical conductivity, Seebeck coefficient, and thermal conductivity of the respective pellets from room temperature until 833 K (**Figure** **5**). The measurements were performed both in the direction parallel and perpendicular to the pressing axis. Here, we exclusively discuss the properties in the parallel direction. Measurements in the perpendicular direction show the same trends and can be found in the Figure S11, Supporting Information.

Na–SnSe has higher electrical conductivity than Me_4_N–SnSe over the whole temperature range. Hall effect measurements revealed that this difference is caused by the three orders of magnitude higher hole concentration of Na–SnSe, ≈2.3 × 10^19^ cm^−3^, than Me_4_N–SnSe, ≈2.8 × 10^16^ cm^−3^. Na is frequently used as a p‐type dopant in SnSe due to its tendency to replace lattice Sn^2^^+^.^[^ ^77^, ^78^, ^79^ ^]^ In Na–SnSe samples, part of the Na diffused into the crystal lattice (Figure 3), either during the particle synthesis or through the consecutive thermal processes to produce the pellet, thus explaining the high carrier concentration measured.

The electrical conductivities of both Na–SnSe and Me_4_N–SnSe show a thermally activated behavior, typical of polycrystalline SnSe.^[^ ^4^, ^27^, ^77^, ^80^ ^]^ We calculated the weighted mobilities (*µ*_W_) from the Seebeck coefficients and electrical conductivities^[^ ^81^ ^]^ to understand the underlying transport phenomenon (Figure S13, Supporting Information). In the low‐temperature range, the increase of mobility with temperature indicates the presence of energy barriers at the grain boundaries.^[^ ^82^, ^83^ ^]^ The barrier height (*E*_b_; σ ∝ *T* ^−1/2^ exp(−*E*_b_/k_B_ *T*)) for the Na–SnSe sample (≈172 meV) is more than double that for the Me_4_N–SnSe (≈75 meV). This is also consistent with Na–SnSe lower hall mobility at room temperature, ≈2.3 cm^2^ V^−1^ s^−1^ versus 21.2 cm^2^ V^−1^ s^−1^ for Me_4_N–SnSe. As the temperature increases, the effect of the potential barriers diminishes due to thermal carrier excitations.

We observed that the Seebeck coefficient of the Na–SnSe samples is higher than those previously reported for polycrystalline samples obtained through melting and annealing, despite the similar charge carrier densities. To illustrate this, we plotted the Seebeck coefficient as a function of charge carrier concentration (Pisarenko plot) at 300 K and compared it with reported experimental data and first‐principles calculations using a multiple band model^[^ ^84^ ^]^ in Figure 5E. The plot reveals that not only our Na–SnSe sample but also many other solution‐processed SnSe (half‐filled green symbols)^[^ ^22^, ^27^, ^29^, ^31^, ^42^, ^43^, ^85^ ^]^ have Seebeck coefficients exceeding the expected value, a tendency not observed in Na‐doped solid‐state synthesized SnSe (black open symbols,^[^ ^47^, ^53^, ^77^, ^78^, ^86^ ^]^ including single crystals^[^ ^87^, ^88^ ^]^).

The high carrier concentration in the Na–SnSe samples comes from the partial diffusion of ionically adsorbed Na into the SnSe crystal lattice during the thermal processing (annealing + spark plasma sintering). However, the amount of Na in the final material cannot be precisely controlled when using Na salts during the particle synthesis, as the Na content is determined by the electrostatic adsorption of Na^+^ ions at the particle surface. The amount of Na introduced through surface adsorbates in Na–SnSe exceeds its solubility limit,^[^ ^53^ ^]^ resulting in partial segregation of Na forming Na‐rich dislocations, grain boundaries, and nanoprecipitates (Figures 3 and 4). Kelvin probe force microscopy (KPFM) measurements of the surface potential across Na‐rich grain boundaries in the Na–SnSe sample revealed charge accumulation and downward band bending at the grains interface (Figure 5F).^[^ ^89^, ^90^ ^]^ These results suggest that the Na‐rich interfaces act as potential barriers,^[^ ^89^, ^90^, ^91^ ^]^ filtering low energy holes and enhancing the Seebeck coefficient.^[^ ^92^, ^93^ ^]^ In contrast, for Me_4_N–SnSe, which has no detectable secondary phases at the grain boundaries (Figure 4B; Figure S8, Supporting Information), the Seebeck coefficient fits the value expected by the calculated Pisarenko relation (Figure 5E).

To better understand the role of excess Na, we developed a doping strategy using Me_4_N–SnSe particles and adding Na after particle purification (details can be found in the Experimental Section). This strategy allows us to control the Na concentration in the final material. We adjusted the content of Na to have carrier concentrations comparable to those obtained for Na–SnSe, but significantly lower content of Na, ≈1% (Figures S14–S16, Supporting Information). The Na‐doped Me_4_N–SnSe sample, named Me_4_N–SnSe + Na shows a trend for the electrical conductivity similar to Na–SnSe, with carrier concentration of ≈10^19^ cm^−3^, yet lower Seebeck coefficient (Figure 5B) at low temperatures. The room temperature Seebeck coefficient of Me_4_N–SnSe +Na fits perfectly with the values expected from the Pisarenko relation calculated using a multiband model (Figure 5E, red rhombus), indicating no energy filtering effects in this sample.

Despite the significant difference in the microstructures, the three samples (Na–SnSe, Me_4_N–SnSe, and Me_4_N–SnSe + Na) have very similar lattice thermal conductivity (Figures S11 and S12, Supporting Information). We attribute this to the strong lattice anharmonicity in SnSe as the dominant effect contributing to the thermal conductivity.^[^ ^88^ ^]^ Overall, we find that the calculated *zT* is doubled for Na–SnSe compared to Me_4_N–SnSe (Figure S12, Supporting Information). By increasing the carrier concentration by carefully adding Na after the Me_4_N–SnSe particle synthesis, the resulting Me_4_N–SnSe + Na sample shows slightly inferior *zT* than Na–SnSe (Figure S12, Supporting Information). We attribute this to a favorable energy filtering effect in Na–SnSe compared to Me_4_N–SnSe + Na (Figure S17, Supporting Information). However, to estimate the ideal degree of filtering, further work is necessary.

## 3 Conclusion

We investigated the role of surface ion adsorbates in polycrystalline SnSe produced from surfactant‐free SnSe particles. We found that when incorporating Na salts in the reaction mixture, Na^+^ ions are electrostatically adsorbed on the particle surface and remain there after the particles are removed from the solution. Moreover, Na remains in the material during the annealing and sintering steps, playing an important role in the microstructure evolution and the final material functional properties. In the sintered pellets, Na is present within the matrix, in dislocations, precipitates, and forming grain boundary complexions. Due to the tendency of Na^+^ to occupy Sn^2+^ sites, Na–SnSe samples exhibit high carrier concentrations. Moreover, the interface between Na‐rich segregates and SnSe grains induces energy barriers, leading to charge carrier energy filtering and enhancing the Seebeck coefficient.

This work reveals the presence of surface adsorbates in solution‐processed surfactant‐free methods and their effect on the transport properties. Furthermore, we explain the rationale behind their presence based on the fundamentals of colloidal science. These findings are relevant because they go beyond the specific system studied. They highlight the importance of evaluating possible unintentional impurities and their origin to i) establish the proper structure–property relationships and ii) redefine synthetic protocols to tune material properties controllably.

An example where the lack of impurity awareness has limited our capability to optimize material properties is n‐type SnSe. Contrary to p‐type, where solution methods have reached state‐of‐the‐art thermoelectric performance at high temperature, for n‐type SnSe, *zT* is much lower than half of the highest reported value (*zT* of 1.1 for solution‐processed material compared to 2.8 for single crystal).^[^ ^94^, ^95^ ^]^ We believe this is correlated to the fact that all synthetic methods use Na salts to produce SnSe particles. Hence, when n‐type dopants are introduced, the presence of Na can establish a pinning problem.

## 4 Experimental Section

### SnSe Particle Synthesis

SnSe particles were prepared following a procedure previously reported by Han et al.^[^ ^33^ ^]^ with slight modifications. In a typical synthesis, NaBH_4_ (160 mmol, 98%, Fisher Scientific) was first dissolved in 400 mL deionized water, and then Se powder (80 mmol, 100 mesh, ≥99.5%, Sigma–Aldrich) was slowly added into the solution. Stirring should be avoided during this step due to the strong evolution of hydrogen gas. Once the bubbling finished, stirring was resumed under Ar flow until the solution became transparent indicating the complete reduction of Se. In parallel, NaOH (750 mmol, 98% Fisher Scientific) and SnCl_2_ **·**2H_2_O (72 mmol, 98%, Fisher Scientific) were mixed with 360 mL of deionized water. The mixture was stirred at room temperature under Ar flow until complete dissolution. At this point, the solution was heated under reflux to its boiling point (≈101.3 °C). The freshly prepared selenide solution was rapidly injected into the boiling tin solution, and the temperature dropped to ≈70 °C. Upon injection, the reaction mixture turned black indicating the particle formation. The temperature was allowed to recover to 101.3 °C and this temperature was maintained for 2 h. To purify the as‐synthetized particles, the mixture was decanted, and the transparent supernatant was carefully discarded. The remaining crude mixture (≈120 mL) was transferred into centrifuge tubes. The particles were purified by three precipitation/re‐dispersion cycles with ≈100 mL of deionized water and ethanol alternatively. In the first cycle, the fresh deionized water was added into the crude solution by centrifugation at 6000 rpm for 1 min. Then, ethanol was used to redisperse the particles and the solution was centrifuged at 8000 rpm for 5 min. In the second cycle, deionized water was added to solubilize the remaining impurities and particles were precipitated by centrifugation at 9000 rpm for 5 min. Afterward, ethanol was employed to re‐disperse and precipitate the particles by centrifugation at 8000 rpm for 5 min. These same steps were repeated for a third purification cycle. Washed particles were dried under vacuum overnight at room temperature and kept in the glovebox for further use.

### Me_4_N–SnSe Particle Synthesis

SnSe particles without Na were produced following the above procedure keeping the molar ratios and using (CH_3_)_4_NOH**·**5H_2_O (tetramethylammonium hydroxide pentahydrate, 98%, Sigma‐Aldrich) and (CH_3_)_4_NBH_4_ (tetramethylammonium borohydride, 95%, Sigma–Aldrich) instead of the corresponding Na‐reagents.

### Me_4_N–SnSe + Na Sample Preparation

As‐synthesized Me_4_N–SnSe particles were first annealed at 550 °C for 1 h under a forming gas (95% N_2_ + 5% H_2_) flow inside a tube furnace with the heating rate of ≈10 °C min^−1^. Subsequently, the annealed powder was finely ground with an agate mortar in a glovebox. Afterward, 4 g of annealed Me_4_N–SnSe powder was mixed with 5.5 mL CH_3_COONa (99%, Sigma–Aldrich) solution (3 mg mL^−1^ in methanol) and vigorously stirred overnight at room temperature, and then dried under vacuum.

### Thermal Processing

Dried SnSe particles (both of Na–SnSe and Me_4_N–SnSe) were annealed at 500 ^°^C for 1 h under a forming gas (95% N_2_ + 5% H_2_) flow inside a tube furnace with the heating rate of ≈10 °C min^−1^. Then, the annealed was powder finely ground with an agate mortar and loaded into a graphite die in a glovebox before being pressed into cylinders (Ø 8.6 mm × 12 mm). The process was carried out under vacuum, in an AGUS PECS Spark Plasma Sintering (SPS) System – Model SPS 210S× applying a pressure of 45 MPa and at temperature of 500 °C for 5 min. For preparation of Me_4_N–SnSe + Na pellet: dried Me_4_N–SnSe + Na composites were annealed at 550 °C for 3 h under a forming gas flow. Then, the powder was consolidated into a cylinder under an axial pressure of 45 MPa at temperature of 550 °C for 10 min. The relative densities of the compacted pellets were measured by the Archimedes′ method and found to be ≈92–95% of the theoretical value in all samples. From these cylinders, round shaped pellets and rectangular bars were cut in two normal directions, that is, parallel to the pressing direction and within the cylinder plane.

### Structural and Chemical Characterization

X‐ray diffraction (XRD, 2θ: 20° to 60°; scanning rate: 5° min^−1^) analyses were carried out on a AXS D8 ADVANCE (Bruker) X‐ray diffractometer with Cu K_α_ radiation (λ = 1.5406 Å). Size and morphology of initial particles, annealed nanopowders, and sintered pellets were examined by field‐emission scanning electron microscopy (SEM) on a Merlin VP Compact (Zeiss) operated at 5.0 kV. Composition was investigated by using a EDAX Octane Elite energy dispersive X‐ray spectrometer (EDS) attached to the Merlin VP Compact and operated at 15.0 kV. The material composition was also analyzed by means of ICP‐OES on an ICPE‐9820 (Shimadzu) system, and samples were prepared by the digestion of ground powder in aqua regia overnight followed by dilution in MQ‐Water (Milipore). Zeta potential and Zeta deviations were measured with a Malvern Panalytical Zetasizer Nano (λ = 633 nm) in samples diluted in water (≈0.1 mg mL^−1^, pH = 6.5–7.0). The results are the average of four measurements with standard deviation <1 and were determined using the Smoluchowski approximation.

HRTEM and STEM studies were conducted using a JEM‐2800 (JEOL Ltd.) field emission gun microscope operated at 200 kV equipped with a XF416ES (TVIPS GmbH) for HRTEM images and Centurio large solid angle silicon drift detector (JEOL Ltd.) with 100 mm^2^ active area for STEM–EDS analysis. The lamellae for TEM and STEM studies were prepared by standard “lift‐out” procedure directly from the sintered pellet. The lamella was attached to a gold grid instead of a standard Cu half‐moon grid to avoid overlapping the Na K_α_ line (1.041 keV) and the Cu L_α_ line (0.930 keV). The lamella lift‐out and thinning was carried out on an Aquilos 2 dual beam system (Thermo Fisher Scientific).

XPS was carried out on a Specs system equipped with a Mg anode XR50 source operating at 250 W and a Phoibos 150 MCD‐9 detector (Specs GmbH). The pressure in the analysis chamber was kept below 10^−7^ Pa. Data processing was performed with the CasaXPS program (Casa Software Ltd.). Needle‐shaped APT specimens were prepared by a standard “lift‐out” method in a SEM/FIB dual‐beam focused ion beam microscope (Helios NanoLab 650, FEI).^[^ ^96^ ^]^ APT measurements were performed on a local electrode atom probe (LEAP4000X Si, Cameca) by applying 10‐ps, 5‐pJ ultraviolet (λ = 355 nm) laser pulses at 30 K to minimize surface migration. The pulse repetition rate was 200 kHz, and the detection rate was 1 ion per 100 pulses, and the flight path 160 mm. APT data were processed with the commercial software package IVAS 3.8.0. Samples were polished with a vibratory polishing tool (VibroMet 2). AFM and KPFM measurements were performed in a Park NX20 atomic force microscope equipped with a multiE75_g Tip and operated with a sample bias of 80 mV.

### Thermoelectric Characterization

Seebeck coefficients were measured by using a static DC method. Electrical resistivity data was obtained by a standard four‐probe method. Seebeck coefficient and electrical resistivity were measured simultaneously in an LSR‐3 (Linseis) system between room temperature and 833 K, under Helium atmosphere. Three up and down measurements were performed. Taking into account the system accuracy and the measurement precision, an error of ≈4% in the measurement of both the electrical conductivity and Seebeck coefficient was estimated. The thermal conductivity was calculated by *κ = λ C*_p_ ρ, where λ is the thermal diffusivity, *C*_p_ is the heat capacity, and ρ is the mass density of the specimen. An LFA 1000 (Linseis) was used to determine the thermal diffusivities (λ) of the samples by the Laser Flash method with an estimated error of ≈2.4%. The constant pressure heat capacity (*C*_p_) was estimated from empirical formula by the Dulong–Petit limit (3R law), and the density (ρ) values were measured using the Archimedes′ method. To avoid cluttering the plots, error bars were not included in the figures. Room temperature Hall charge carrier concentrations (*n*_H_) were measured with the Van der Pauw method using a magnetic field of 0.6 T (ezHEMS, NanoMagnetics). Values provided correspond to the average of six measurements, from which an error of ≈15% was estimated.

## Conflict of Interest

The authors declare no conflict of interest.

## Supporting information

Supporting Information

## Data Availability

The data that support the findings of this study are available from the corresponding author upon reasonable request.
