# Supplementary material for: The Importance of Surface Adsorbates in Solution‐Processed Thermoelectric Materials: The Case of SnSe
Source: Adv Mater. 2021 Oct 22;33(52):2106858. doi: 10.1002/adma.202106858 (PMC11468704; doi:10.1002/adma.202106858)
Supplement: Supplementary file 1 — Supporting Information [file ADMA-33-2106858-s001.pdf]

ADVANCED MATERIALS

Supporting Information

for *Adv. Mater.,* DOI: 10.1002/adma.202106858

The Importance of Surface Adsorbates in Solution-Processed Thermoelectric Materials: The Case of SnSe

*Yu Liu, Mariano Calcabrini, Yuan Yu, Aziz Genç, Cheng Chang, Tommaso Costanzo, Tobias Kleinhanns, Seungho Lee, Jordi Llorca, Oana Cojocaru-Mirédin, and Maria Ibáñez**

Supporting Information

The importance of surface adsorbates in solution-processed thermoelectric materials: the case of SnSe

*Yu Liu,[#] Mariano Calcabrini,[#] Yuan Yu, Aziz Genç, Cheng Chang, Tommaso Costanzo, Tobias Kleinhanns, Seungho Lee, Jordi Llorca, Oana Cojocaru-Mirédin, Maria Ibáñez**

Dr. Y. Liu, M. Calcabrini, Dr. C. Chang, Dr. T. Constanzo, T. Kleinhanns, S. Lee, Prof. Maria Ibáñez
IST Austria, Am Campus 1
3400 Klosterneuburg, Austria.
E-mail: mibanez@ist.ac.at

[#] These authors contributed equally to this work.

Dr. Y. Yu, Dr. O. Cojocaru-Mirédin
RWTH Aachen, I. Physikalisches Institut (IA), Sommerfeldstraße 14
52074 Aachen, Germany.

Prof. A. Genç
Department of Materials Science and Engineering, Faculty of Engineering, İzmir Institute of Technology
İzmir 35430, Turkey.

Prof. J. Lorca
Institute of Energy Technologies, Department of Chemical Engineering and Barcelona Research Center in Multiscale Science and Engineering, Universitat Politècnica de Catalunya
08019 Barcelona, Spain.

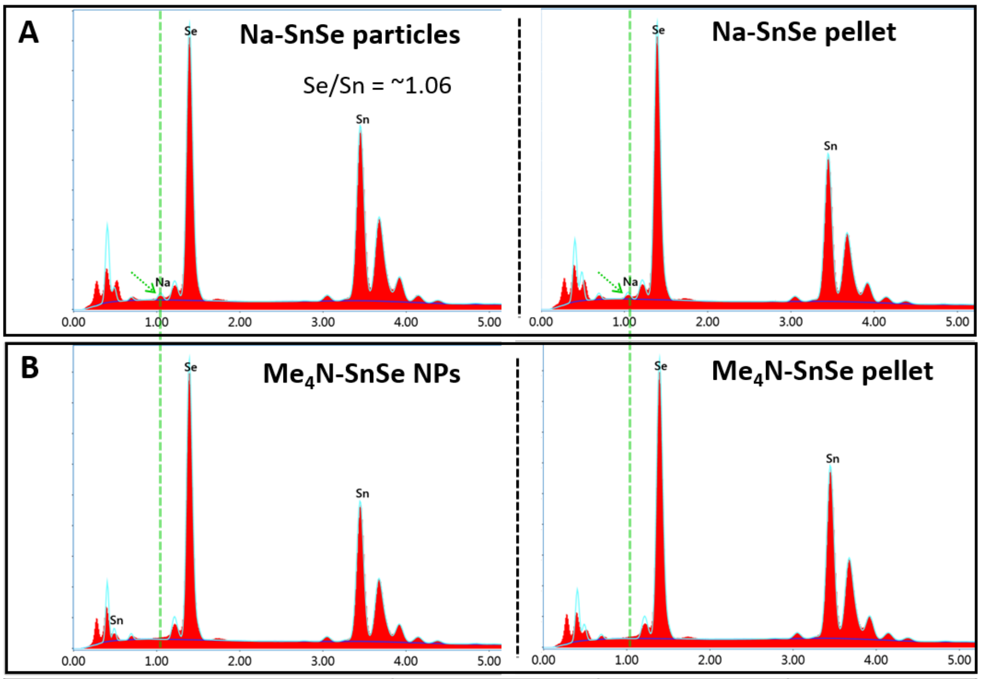

C Na-SnSe	ICP-OES	EDS	XPS
**Atomic ratio (Na/Sn) Particles**	0.0163	0.016	0.085
**Atomic ratio (Na/Sn) Pellet**	0.0155	0.016	*n.a.*

Figure S1. SEM-EDS spectra of (A) as-synthesized Na-SnSe particles and pellet; (B) as-synthesized Me_4N-SnSe particles and pellet; C) Atomic ratio (Na/Sn) of Na-SnSe particles and pellet obtained by ICP-OES, EDS, and XPS. "*n.a.*" refers to not available.

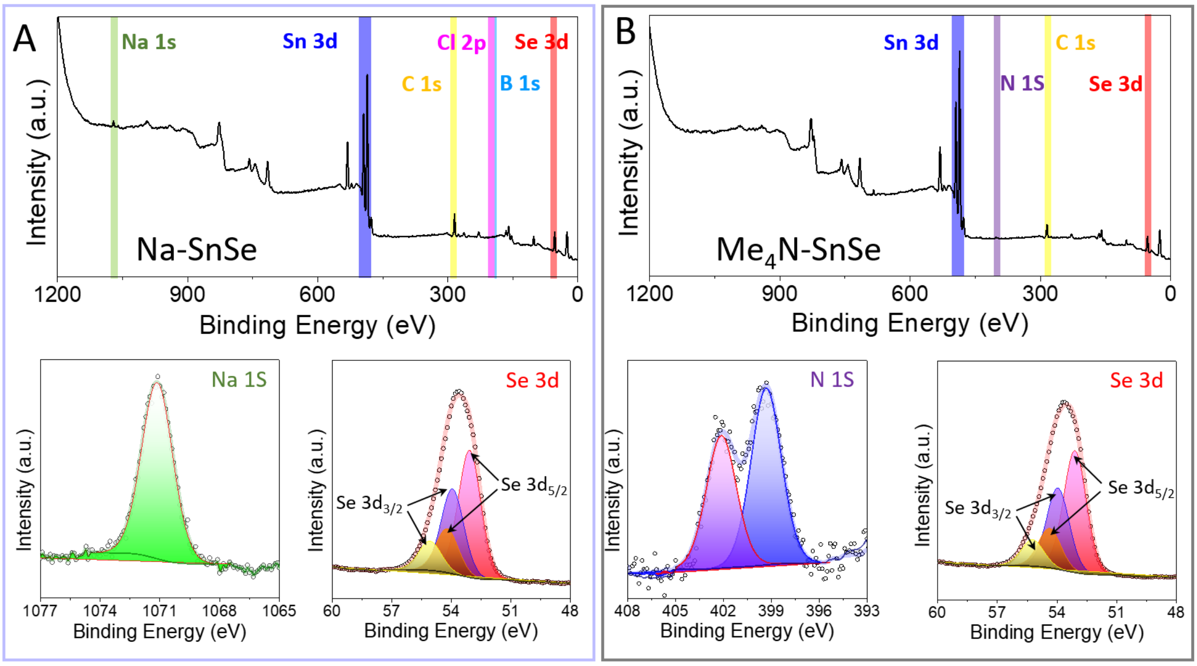

Figure S2. XPS survey spectra of as-synthesized SnSe particles A) Na-SnSe and B) Me_4N-SnSe, respectively, including the high-resolution spectrum of Na 1s, Se 3d for Na-SnSe (A); and N 1s, Se 3d for Me_4N-SnSe (B).

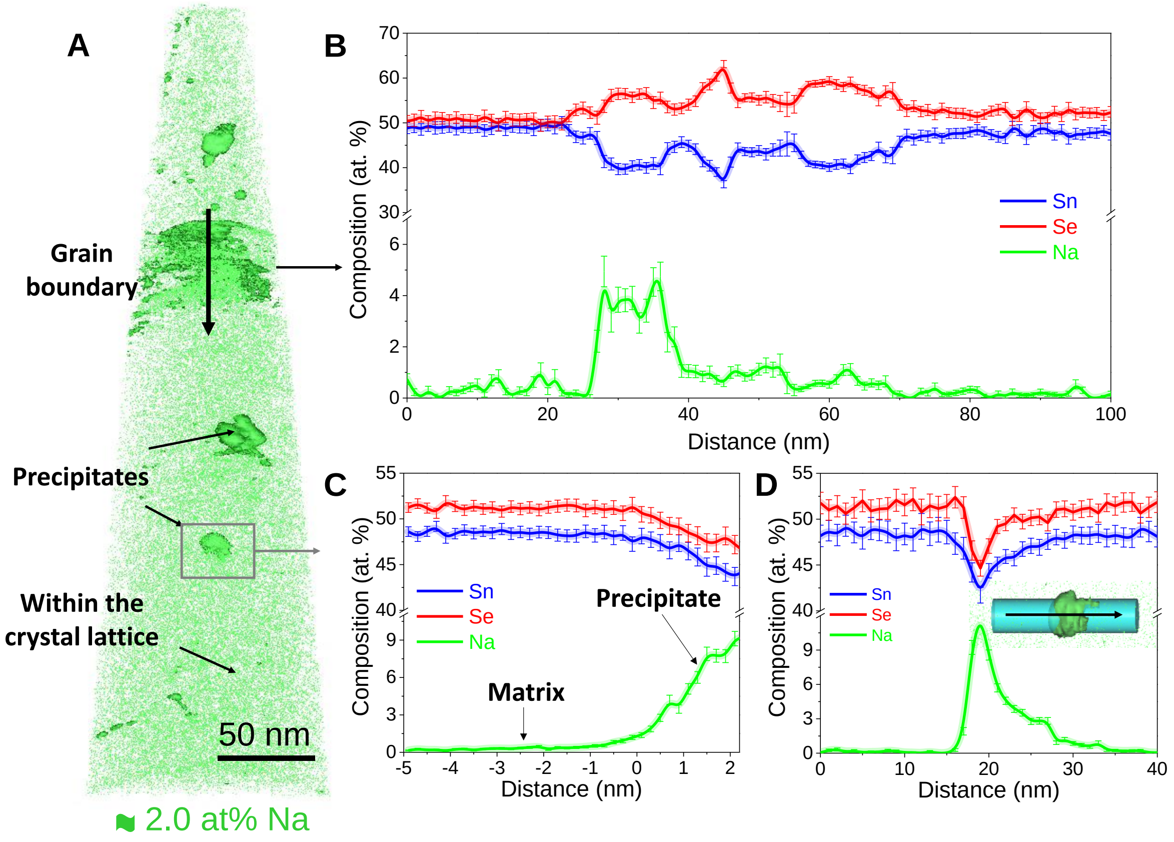

Figure S3. A) Na-rich grain boundary and some precipitates depicted by the isocomposition surface of 2.0 at. % Na.[1] The 1D composition profile across the grain boundary determines that the maximum enrichment of Na at this grain boundary is 5%. Besides, a high concentration of Sn vacancies was also quantified at the grain boundary (B); C) The composition proximity histogram of a precipitate demonstrates the aggregation of Na; D) The nature of Na enrichment is also confirmed by a 1D composition profile across a precipitate.

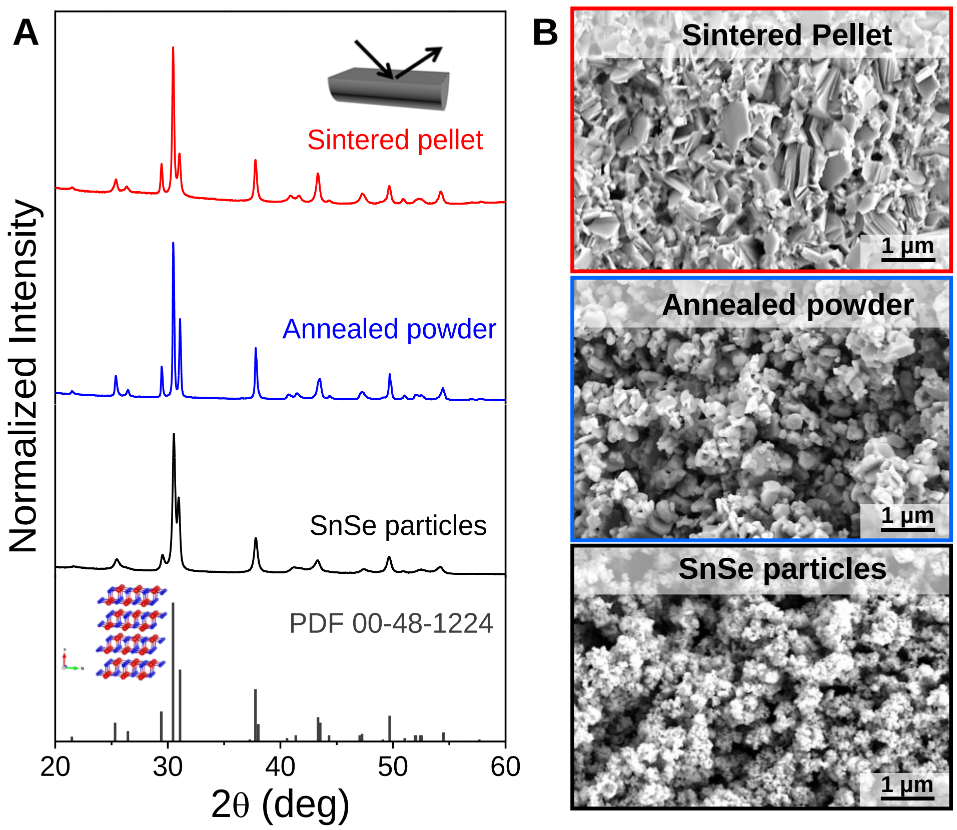

Figure S4. A) XRD patterns of as-synthesized Me_4N-SnSe particles, annealed powder and sintered pellet along out of plane direction including the PDF 00-48-1224 reference. The inset shows the crystal structure of orthorhombic SnSe; B) the corresponding representative SEM micrographs.

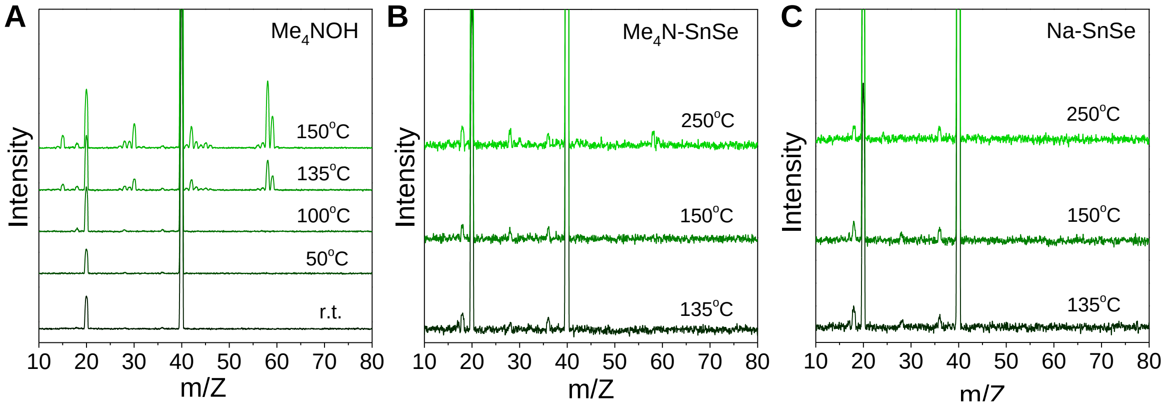

m/Z fragments	Peak assignment	Presence in the samples
Ar = 20, 40, 36	Carrying gas	Present in all samples
N_2 = 28<br>H_2O =18	Adsorbed impurities	Present in all samples
59: $(CH_3)_4N^+$<br>45: $NH(CH_3)_2$<br>46: CH_3OCH_3<br>15: CH_3<br>30: $NHCH_3$	Tetramethylammonium decomposition products	In $(CH_3)_4NOH \cdot 5H_2O$. Dehydration starts at 100°C. Decomposition follows from 135°C.<br>In Me_4N-SnSe nanoparticles the decomposition starts at higher temperature (250°C) because the ions are stabilized by the adsorption on the NP surface.

Figure S5. Headspace mass spectrometry analysis to analyze the decomposition of tetramethylammonium in A) Tetramethylammonium hydroxide (Me_4NOH); B) Me_4N-SnSe particles and C) Na-SnSe particles.

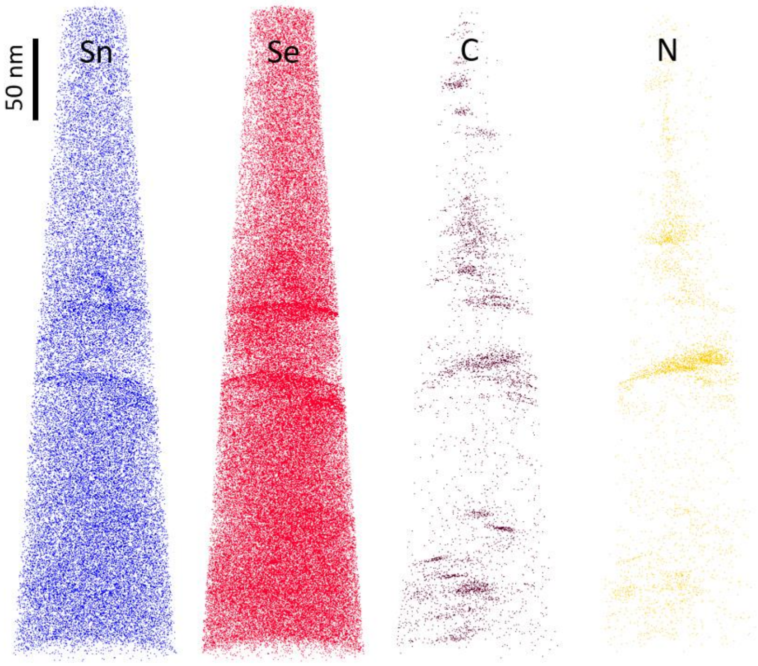

Figure S6. APT of a Me_4N-SnSe pellet showing the 3D distribution of each element. Sn and Se show an almost homogeneous distribution, and very small traces of N and C in the final pellet present aggregations in the grain boundary.

Raman spectroscopy was carried out on a confocal Raman spectrometer (Renishaw inVia Qontor) equipped with a Leica DM2700M microscope over the range of 37 to 2000 cm^{-1}. A laser excitation source of 532 nm and a grating of 2400 lines mm^{-1} were used. For each analysis 12 spectra were acquired with an exposure time of 1 s and a laser power of 1 mW cm^{-2}.

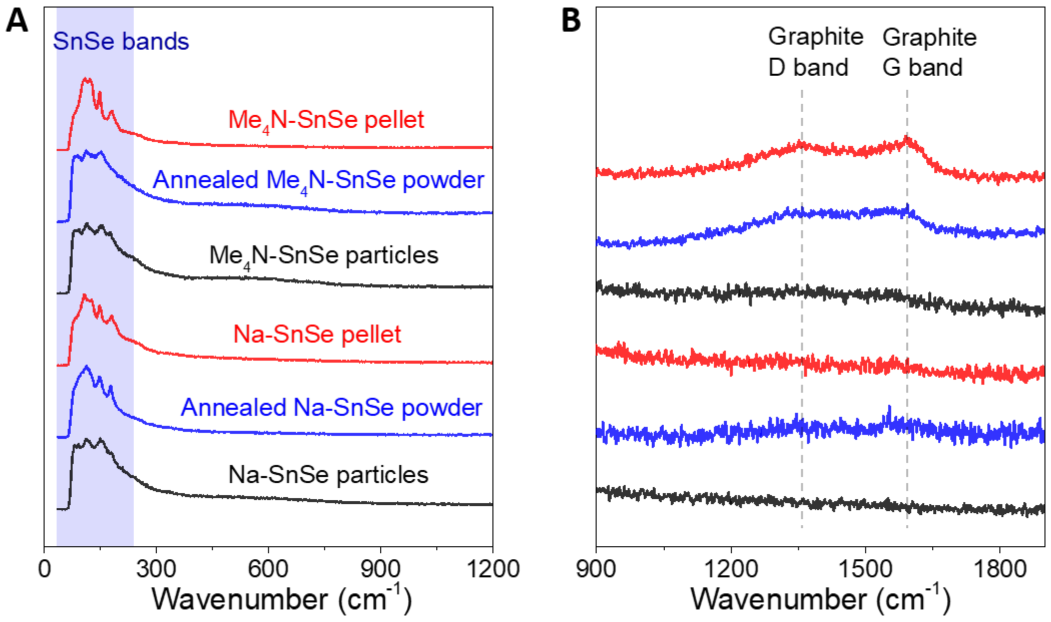

Figure S7. A) Raman spectrum of as-synthesized SnSe, Me_4N-SnSe particles, annealed SnSe, Me_4N-SnSe powders and SnSe, Me_4N-SnSe pellets, respectively. B) Magnification of the Raman pattern in the region of 900-1900 cm^{-1}.

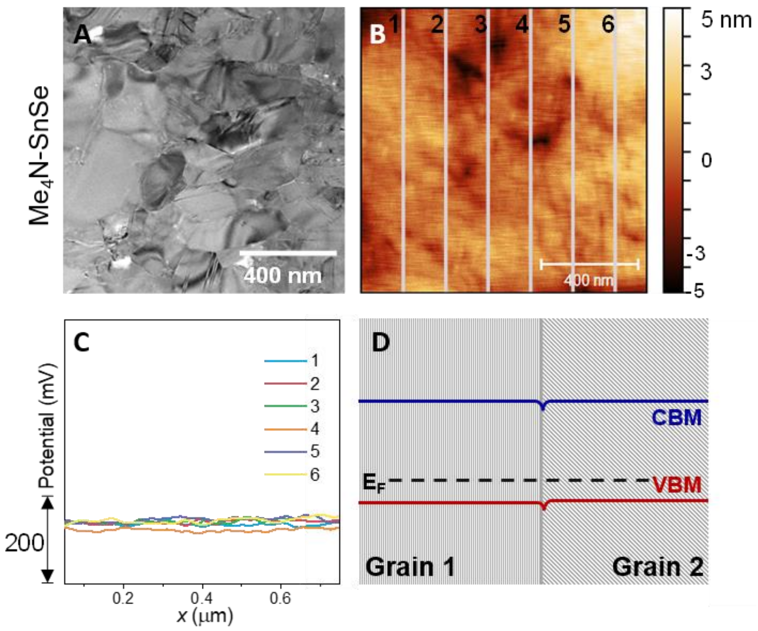

Figure S8. A) STEM image, B) AFM topology maps, C) KPFM potential profiles across the lines indicated in the AFM topology map, and D) scheme showing the band alignment at the grain boundary in Me_4N-SnSe. CBM, VBM, and E_F indicate the conduction band minimum, valence band maximum, and Fermi level, respectively.

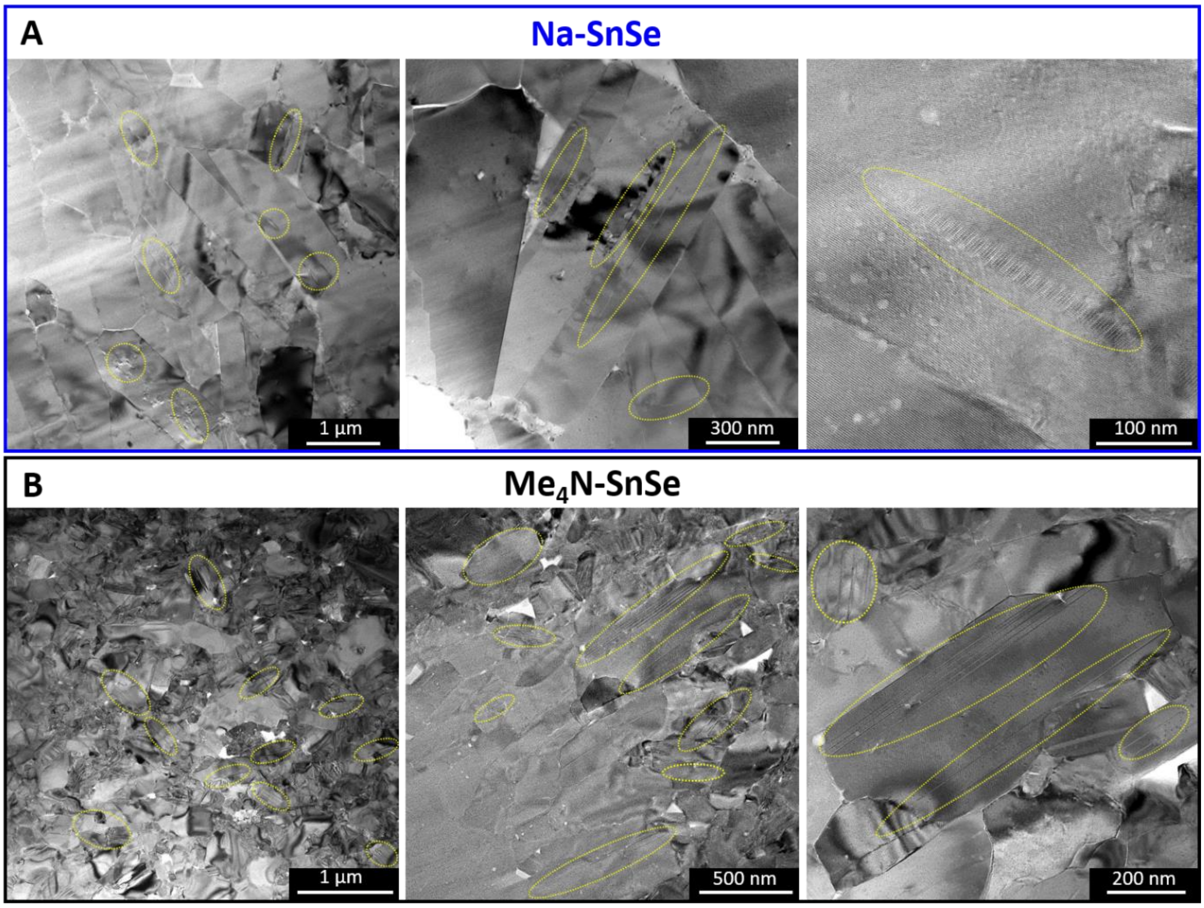

Figure S9. TEM images of SnSe pellets indicating dislocations in A) Na-SnSe and B) Me_4N-SnSe.

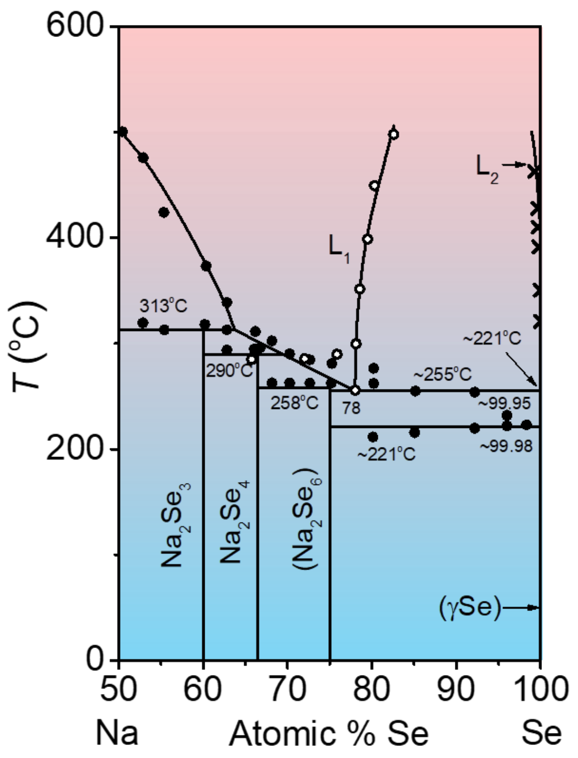

Figure S10. Na-Se phase diagram.[2]

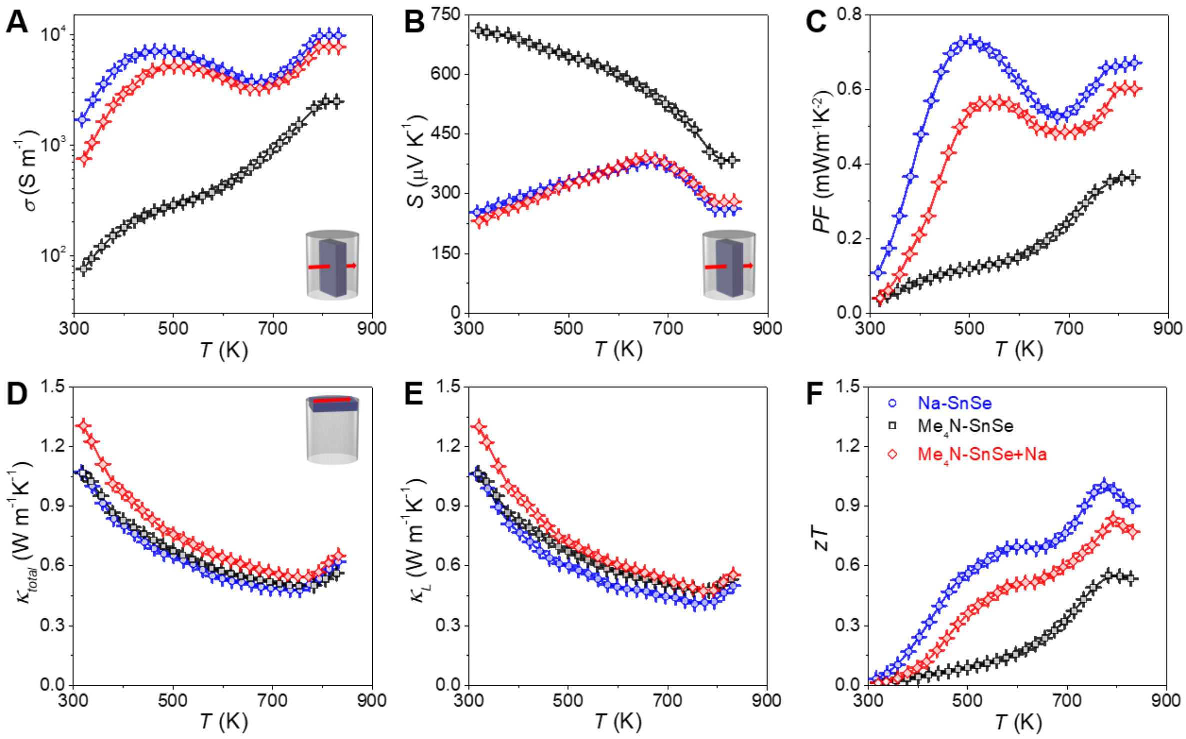

Figure S11. Thermoelectric properties of Na-SnSe (blue), Me_4N-SnSe (black), and Me_4N-SnSe+Na (red) samples measured in perpendicular direction: A) electrical conductivity, σ; B) Seebeck coefficient, S; C) power factor, PF; D) thermal conductivity, κ_{total}; E) lattice thermal conductivity, κ_L; and F) figure-of-merit, zT.

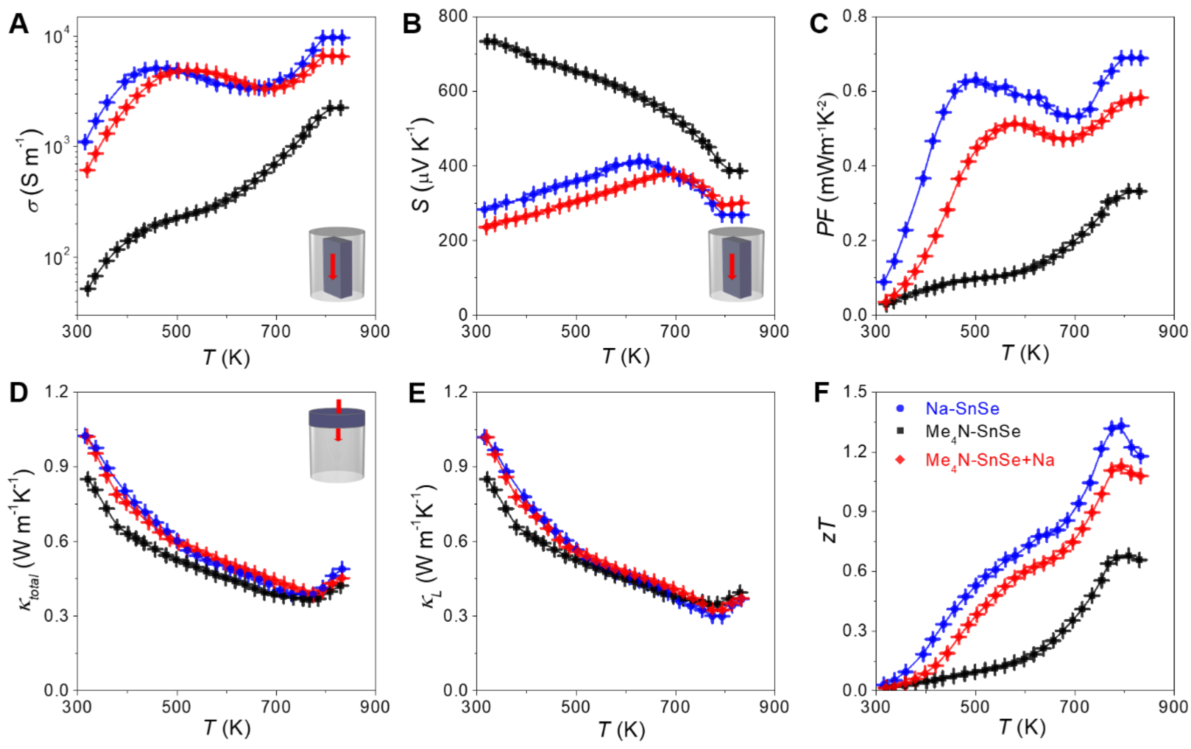

Figure S12. Thermoelectric properties of Na-SnSe (blue) and Me_4N-SnSe (black), and Me_4N-SnSe+Na (red) samples measured in parallel direction: A) electrical conductivity, σ; B) Seebeck coefficient, S; C) power factor, PF; D) thermal conductivity, κ_{total}; E) lattice thermal conductivity, κ_L; and F) figure-of-merit, zT.

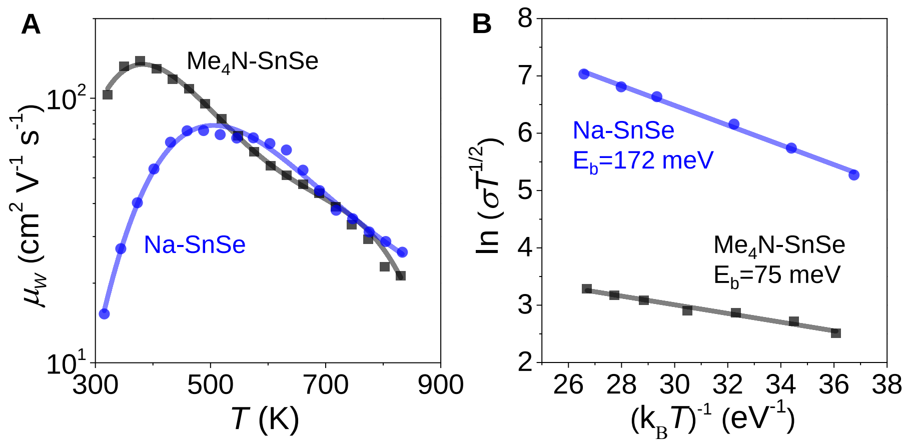

Figure S13. A) Weighted mobility, μ_W; B) energy barrier, E_b ($\sigma \propto T^{-1/2} \exp(-E_b/k_BT)$) of Na-SnSe and Me_4N-SnSe sample.

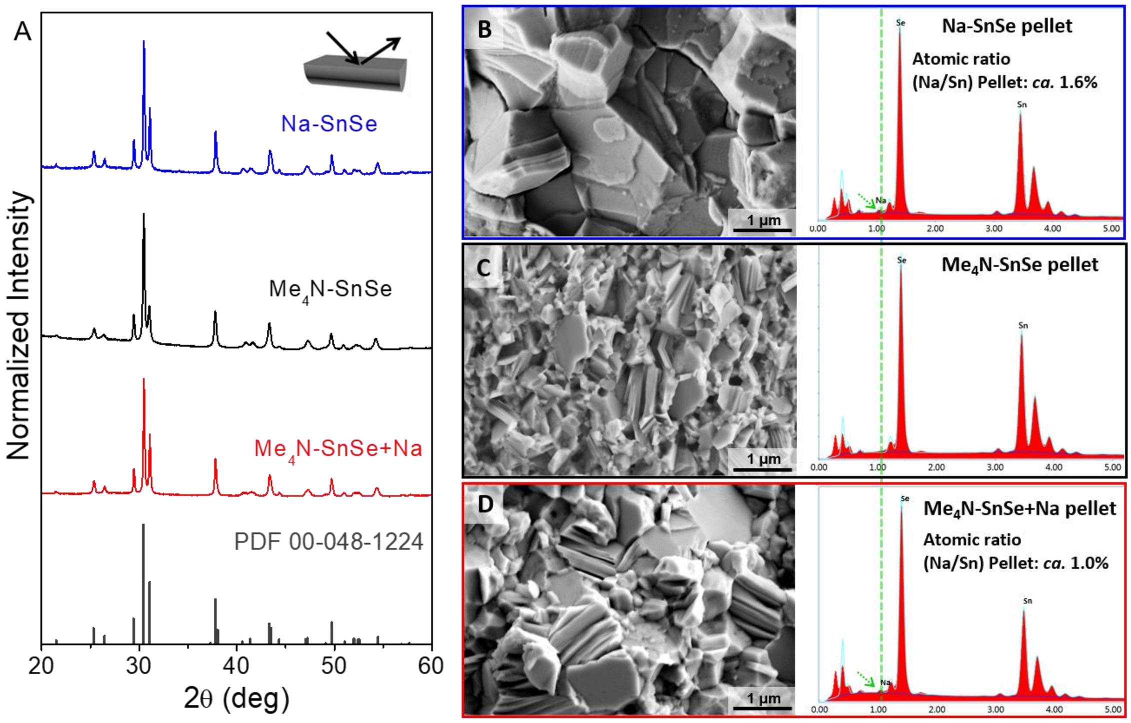

Figure S14. A) XRD patterns of Me_4N-SnSe+Na, Me_4N-SnSe, and Na-SnSe along out of plane direction including the PDF 00-48-1224 reference; B)-D) the corresponding representative SEM micrographs and SEM-EDS spectra.

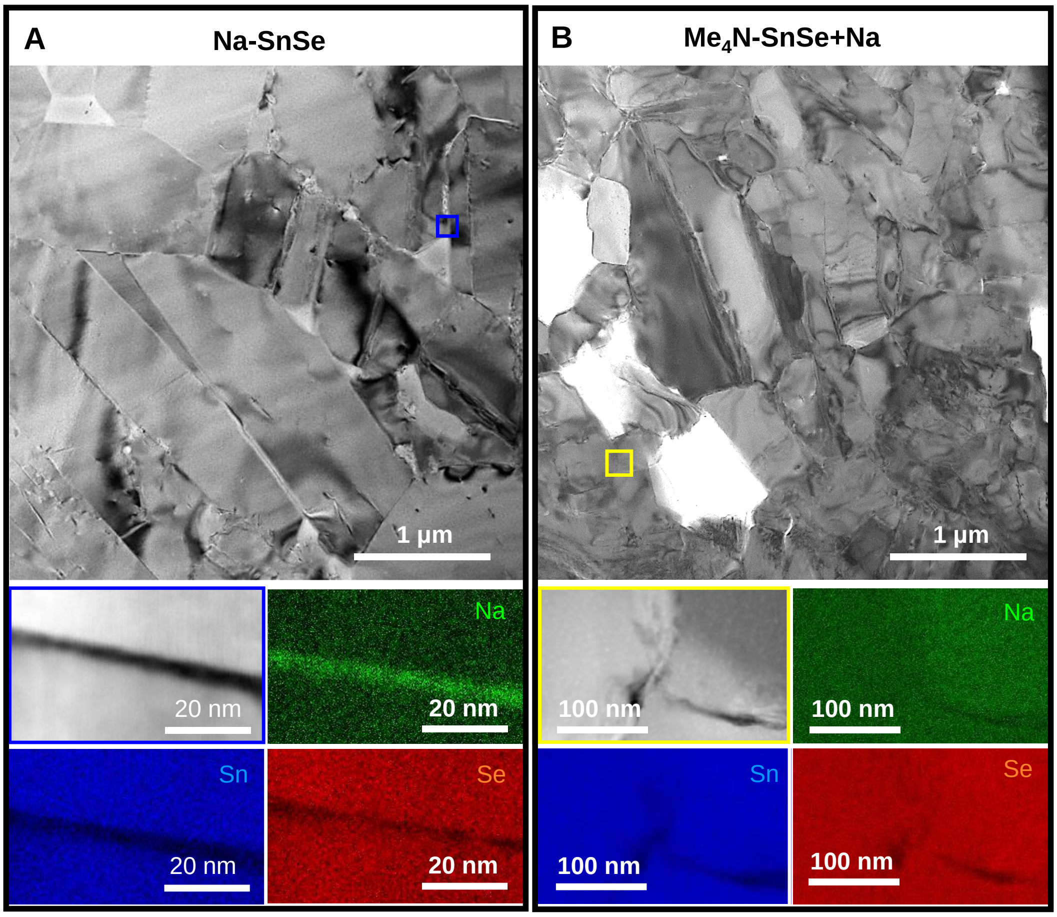

Figure S15. TEM images representing cross-sections of SnSe pellets. General view (top), together with the corresponding STEM-EDS elemental mapping of a grain boundary (bottom) for: A) Na-SnSe; and B) Me_4N-SnSe+Na.

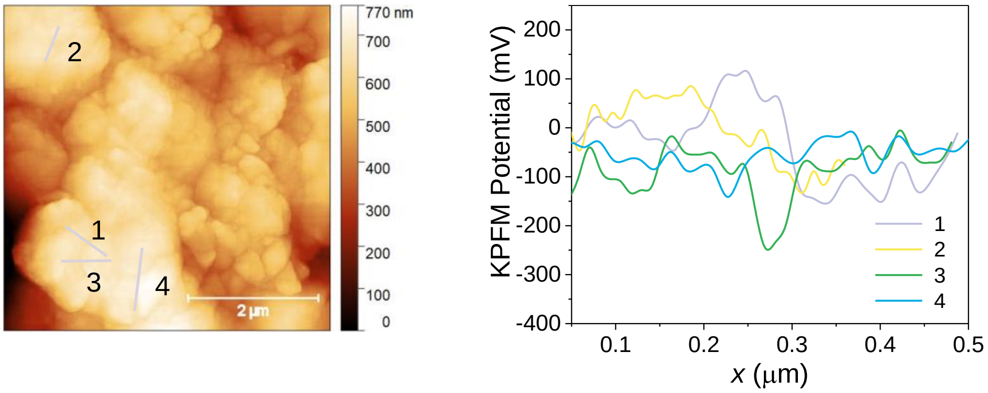

Figure S16. Atom probe microscopy topology maps, Kelvin probe force microscopy potential profiles across the lines indicated in the AFM topology map in Me_4N-SnSe+Na pellet.

To quantify the effect of the grain boundaries on thermal and electronic transport, we utilized a heterogeneous transport model developed recently by Snyder *et. al.*[3]

We calculated the interfacial heat resistance ($\rho_{Kaptiza}$) using the following formula, that considers the grain size (d), the thermal conductivity of the sample (κ_{total}) and the contribution of the grains (excluding boundaries) to the thermal conductivity (κ_{grain}). To determine this last one, we considered the thermal conductivity of a solid-state Na-doped SnSe sample with a similar charge carrier concentration.[4]

$$\rho_{Kapitza} = d\,(\frac{1}{\kappa_{total}} - \frac{1}{\kappa_{grains}})$$

In a similar way, we determined the interfacial electrical resistivity ($\rho_{el\text{-}gb}$) from the electrical conductivity (σ_{total}) and the electrical conductivity of the grains (σ_{grain}):

$$\rho_{el-GB} = d\,(\frac{1}{\sigma_{total}} - \frac{1}{\sigma_{grains}})$$

Finally, we calculated the Seebeck coefficient of the grain boundaries according to the equation:

$$S_{GB} = \frac{S_{total}\,\kappa_{grain} - S_{grain}\kappa_{total}}{\kappa_{grains} - \kappa_{total}}$$

According to this two-phase model, the contribution of the grain boundaries to the figure of merit can be expressed as:

$$zT_{GB} = S_{GB}^2 T\,.\frac{\rho_{Kapitza}}{\rho_{el-GB}}$$

Given that the Seebeck coefficient of the grain boundaries (S_{GB}) is greater than the sample's Seebeck coefficient over the whole temperature range, the ration between the interfacial resistances $\frac{\rho_{Kapitza}}{\rho_{el-GB}}$ determines if the total effect of energy filtering is beneficial for the materials performance.

We performed these calculations both for the Na-SnSe sample and for the Na-doped Me_4N-SnSe sample (low sodium content), and verified that energy filtering has an overall beneficial effect in Na-containing, solution-processed SnSe.

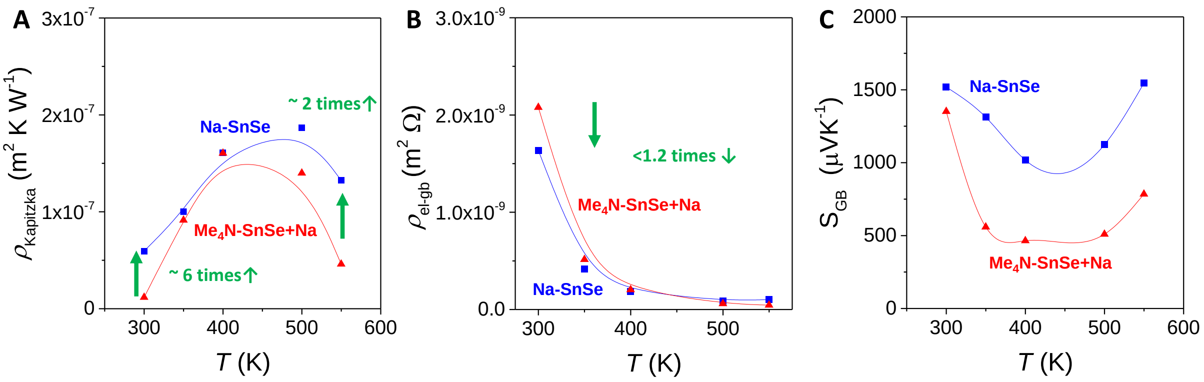

Figure S17. Interfacial heat resistance ($\rho_{Kaptiza}$), interfacial electrical resistivity ($\rho_{el\text{-}gb}$), and interfacial Seebeck coefficient (S_{GB}) for the samples Na-SnSe and Me_4N-SnSe+Na, with comparable charge carrier concentration but very different sodium content. The enhanced interfacial Seebeck coefficient together with the strong enhancement in the heat resistance overcome the effects of the interfacial electrical resistivity in the samples, leading to a beneficial energy filtering effect.

Weight mobility calculation

Weighted mobilities were calculated from electrical conductivity (σ) and Seebeck coefficient (S) measurements according to the equation:[5]

$$\mu_W = \frac{3h^3\sigma}{8\pi e(2m_e k_B T)^{3/2}}\left[\frac{exp\left[\frac{e|S|}{k_B}-2\right]}{1+exp\left[\frac{-5e|S|}{k_B}+5\right]}+\frac{\frac{3}{\pi^2}\frac{e|S|}{k_B}}{1+exp\left[\frac{5e|S|}{k_B}-5\right]}\right]$$

In the above equation σ, S, h, κ_B, e and m_e, are the electrical conductivity, Seebeck coefficient, Plank's constant, Boltzmann's constant, electron charge and electrons mass.

Table S1. Room temperature Hall charge carrier concentration (*n_H*) and Seebeck coefficients (*S*) of Na-SnSe, Me_4N-SnSe and Me_4N-SnSe+Na pellets of three different samples (Figure 5E in the manuscript). Each value of charge carrier concentration in the table corresponds to the average of 6 consecutive measurements with a standard deviation of around 15%. Seebeck coefficients correspond to the average of 3 up and down measurements with an error of *ca.* 4%.

	Sample	n_H (cm^{-3})	Average n_H (cm^{-3})	S ($\mu V\ K^{-1}$)	Average S ($\mu V\ K^{-1}$)
**Na-SnSe**	1	2.5×10^{19}	*ca.* 2.3×10^{19}	262	265
	2	2.1×10^{19}		272	
	3	2.4×10^{19}		261	
**Me_4N-SnSe**	1	3.3×10^{16}	*ca.* 2.8×10^{16}	716	710
	2	2.4×10^{16}		705	
	3	2.7×10^{16}		709	
**Me_4N-SnSe+Na**	1	1.2×10^{19}	*ca.* 1.25×10^{19}	219	226
	2	1.2×10^{19}		229	
	3	1.4×10^{19}		230	

References

1. Y. Liu, M. Calcabrini, Y. Yu, S. Lee, C. Chang, J. David, T. Ghosh, M. C. Spadaro, C. Xie, O. Cojocaru-Mirédin, J. Arbiol, M. Ibáñez, *ACS Nano* **2021**, DOI 10.1021/acsnano.1c06720.
2. J. Sangster; A. D. Pelton. *Journal of Phase Equilibria*. ASM International 1997, pp 185–189.
3. Y. Lin; M. Wood; K. Imasato; J. J. Kuo; D. Lam; A. N. Mortazavi; T. J. Slade; S. A. Hodge; K. Xi; M. G. Kanatzidis; et al. *Energy Environ. Sci.* **2020**, *13*, 4114–4121.
4. Y. Luo; S. Cai; X. Hua; H. Chen; Q. Liang; C. Du; Y. Zheng; J. Shen; J. Xu; C. Wolverton; et al. *Adv. Energy Mater.* **2019**, *9*, 1803072.
5. G. J. Snyder; A. H. Snyder; M. Wood; R. Gurunathan; B. H. Snyder; C. Niu. *Adv. Mater.* **2020**, *32*, 2001537.
